# Familial Hypertrophic Cardiomyopathy Related Cardiac Troponin C L29Q Mutation Alters Length-Dependent Activation and Functional Effects of Phosphomimetic Troponin I*

**DOI:** 10.1371/journal.pone.0079363

**Published:** 2013-11-18

**Authors:** Alison Y. Li, Charles M. Stevens, Bo Liang, Kaveh Rayani, Sean Little, Jonathan Davis, Glen F. Tibbits

**Affiliations:** 1 Biomedical Physiology and Kinesiology, Simon Fraser University, Burnaby, British Columbia, Canada; 2 Molecular Biology and Biochemistry, Simon Fraser University, Burnaby, British Columbia, Canada; 3 Cardiovascular Sciences, Child and Family Research Institute, Vancouver, British Columbia, Canada; 4 Physiology and Cell Biology, The Ohio State University, Columbia, Ohio, United States of America; Tokyo Medical and Dental University, Japan

## Abstract

The Ca^2+^ binding properties of the FHC-associated cardiac troponin C (cTnC) mutation L29Q were examined in isolated cTnC, troponin complexes, reconstituted thin filament preparations, and skinned cardiomyocytes. While higher Ca^2+^ binding affinity was apparent for the L29Q mutant in isolated cTnC, this phenomenon was not observed in the cTn complex. At the level of the thin filament in the presence of phosphomimetic TnI, L29Q cTnC further reduced the Ca^2+^ affinity by 27% in the steady-state measurement and increased the Ca^2+^ dissociation rate by 20% in the kinetic studies. Molecular dynamics simulations suggest that L29Q destabilizes the conformation of cNTnC in the presence of phosphomimetic cTnI and potentially modulates the Ca^2+^ sensitivity due to the changes of the opening/closing equilibrium of cNTnC. In the skinned cardiomyocyte preparation, L29Q cTnC increased Ca^2+^ sensitivity in a highly sarcomere length (SL)-dependent manner. The well-established reduction of Ca^2+^ sensitivity by phosphomimetic cTnI was diminished by 68% in the presence of the mutation and it also depressed the SL-dependent increase in myofilament Ca^2+^ sensitivity. This might result from its modified interaction with cTnI which altered the feedback effects of cross-bridges on the L29Q cTnC-cTnI-Tm complex. This study demonstrates that the L29Q mutation alters the contractility and the functional effects of the phosphomimetic cTnI in both thin filament and single skinned cardiomyocytes and importantly that this effect is highly sarcomere length dependent.

## Introduction

The cardiac troponin (cTn) trimeric complex plays an essential role in regulating cardiac contractility. It consists of a highly conserved Ca^2+^ binding subunit (cTnC) [1], an inhibitory subunit (cTnI), and a tropomyosin binding subunit (cTnT). Together with actin and tropomyosin (Tm), the cTn complex comprises the thin filament, which interacts with the thick filament during contraction. During the diastolic interval, the cTnI-Tm interaction occludes myosin binding sites on actin, and therefore acts as a regulatory switch which prevents cross-bridge formation between actin and the myosin subfragment-1 (S1). Ca^2+^ binding to cTnC triggers conformational changes in the cTnI-actin interaction and other thin filament proteins. These changes produce strong, force-generating interactions between actin and myosin. The Ca^2+^-induced disruption of the cTnI-actin interaction is modulated by the PKA phosphorylation of cTnI, feedback from strong actin-myosin interaction and is also is highly dependent on the sarcomere length [2].

Mutations within the cTn complex can alter both contraction and relaxation of the heart and may lead to cardiomyopathies [3]. Familial hypertrophic cardiomyopathy (FHC) is the most commonly inherited cardiovascular disease with a prevalence of 1∶500 individuals [4] and is the most common cause of sudden cardiac death in young athletes [5]. FHC has been associated with >1000 mutations in ∼20 different sarcomeric and related genes with ∼60 mutations occurring within the cTn complex [3], [6]. The clinical manifestation varies widely, ranging from asymptomatic to severe, including sudden cardiac death caused commonly by arrhythmias and sometimes diastolic dysfunction [4]. Most of the cTn FHC-related mutations identified to date are located within the cTnT and cTnI subunits [3]. The L29Q cTnC mutation, the focus of this work, is the first FHC-associated mutation discovered in cTnC [7]. It should be noted that only one proband with this mutation has been documented to date and the phenotype is not strong and the penetrance is unknown.

Several studies have investigated the molecular mechanisms of this mutation [8]–[14]; however the results have been divergent and the mechanisms by which the L29Q cTnC mutation affects cTnC Ca^2+^ binding remain unclear. We previously demonstrated that the L29Q substitution occurs naturally in cold water fish, and postulated that along with three other teleost-specific substitutions (Asn2, Ile28, and Asp30), they play a role in the higher Ca^2+^ sensitivity involved in adaptation to lower core temperatures [15], [16]. We have also demonstrated that the L29Q cTnC mutation can attenuate the length dependence of contractile force development [12]. The L29Q cTnC mutation may alter the interaction between cTnC and the cardiac-specific N-terminal region of cTnI (residues 1–32) which contain the PKA phosphorylation sites (Ser23/Ser24) [8], [11]. There is also evidence that the L29Q cTnC mutation impacts the interaction between the N-terminus of cTnI and the regulatory domain of cTnC and therefore influences the modulatory effect of cTnI phosphorylation on the cTnI-actin interaction [8], [9], [11], [17], [18].

This study investigates whether the L29Q cTnC mutation abrogates the effects of PKA-dependent phosphorylation of cTnI on cTnC Ca^2+^ binding properties and myofilament Ca^2+^ sensitivity of cTn in multiple systems with increasing complexity. These include cTn complexes, reconstituted thin filaments and ultimately, single skinned cardiomyocytes with the native cTn complexes replaced by recombinant cTn complexes. Different cTn complexes that were used include: mouse wild type cTnC or mutant L29Q cTnC, wild type cTnT, and WT cTnI or SD cTnI. Molecular dynamics simulations were also performed to model the structural changes in the WT & L29Q cTnC/cTnI complex in response to the phosphorylation of the cTnI_1–32_ cardiac specific extension.

The experiments presented herein revealed that the effect of the L29Q mutation varies with the context of the experiments. This variability suggests a greater degree of complexity in cTn function than has been encapsulated in previous work. We show here that the neither the L29Q mutation nor pseudo-phosphorylation has any significant impact on the cTnI/cTnC complex when it is isolated from the thin filament. However, when the L29Q cTnC is incorporated into thin filament preparations the L29Q mutation decreased cTnC Ca^2+^ affinity and in combination with pseudo-phosphorylation of cTnI, extends this effect. When measured in the context of skinned cardiomyocytes, the L29Q mutation yields a small increase in Ca^2+^sensitivity of force, but appears to reduce the effect of phosphomimetic mutations. This effect is most pronounced at shorter sarcomere length (SL), yet it is abrogated with the presence of pseudo-phosphorylation of the cTnI N-terminal extension.

### Abbreviations

See Table 1. Abbreviations: cTn, cardiac troponin; cTnC, cardiac troponin C; TF, thin filament; SL, sarcomere length; SD cTnI, S23D/S24D cardiac troponin I.

**Table 1:** Nomenclature for the recombinant mouse cardiac troponin subunits and troponin complexes used in various preparations.

Recombinant mouse constructs	
WT cTnC	Wild type cTnC
L29Q cTnC	Mutant L29Q cTnC
WT cTnC^T53C^	Wild type cTnC^T53C^-IAANS
L29Q cTnC^T53C^	Mutant L29Q cTnC^T53C^-IAANS
WT cTnI	Wild type cTnI
SD cTnI	Phosphomimetic cTnI (S23/24D)
WT cTnT	Wild type cTnT2
**Troponin Complexes for skinned cardiac myocytes (SCM)**	
WT cTn	WT cTnT, WT cTnI, WT cTnC
WT cTnC^T53C^	WT cTnT, WT cTnI, WT cTnC^T53C^-IAANS
L29Q cTn	WT cTnT, WT cTnI, L29Q cTnC
L29Q cTnC^T53C^	WT cTnT, WT cTnI, Mutant L29Q cTnC^T53C^-IAANS
SD cTn	WT cTnT, S23/24D cTnI, WT cTnC
L29Q SD cTn	WT cTnT, S23/24D cTnI, L29Q cTnC
**Troponin complexes for steady-state and stopped-flow kinetic fluorescence measurements in thin filament preparations (TFP)**	
WT cTn^T53C^	WT cTnT, WT cTnI, WT cTnC^T53C^-IAANS
L29Q cTn^T53C^	WT cTnT, WT cTnI, L29Q cTnC^T53C^-IAANS
SD cTn^T53C^	WT cTnT, S23/24D cTnI, WT cTnC^T53C^-IAANS
L29Q SD cTn^ T53C^	WT cTnT, S23/24D cTnI, L29Q cTnC^T53C^-IAANS

Mouse cTnT2 isoform (GenBank: AAA85350.1) was used for all recombinant Tn complexes.

## Experimental Procedures

### Protein Over-expression and Purification of cTnC, cTnI, and cTnT

Recombinant mouse cardiac protein subunits were expressed in *E. coli*. BL21(DE3) (Novagen) cells. The cTnC gene was in the ampicillin resistant pET21a vector, cTnI and cTnT were in in the kanamycin resistant pSBET and pET24a vectors respectively. Expression and purification of cTnC, cTnI, and cTnT were carried out using methods described in Dong *et al.* and Kruger *et al.* [19], [20].

The method for cTnI was modified as follows: The TnI-containing cell lysate was made to 25% saturation with ammonium sulphate, and subject to centrifugation at 27,000 g for 30 mins (Beckman JLA16.25) at 4°C. The supernatant was dialysed overnight against 2–3 changes of 4 liters of buffer A (in mM: 20 TEA-HCl pH 7.5, 6000 urea, 1 EDTA, and 1 DTT) and loaded onto a CM sepharose column (XK26, Amersham Biosciences) previously equilibrated with buffer B (in mM: 20 Tris-HCl, 6000 urea, 1 EDTA, and 1 DTT at pH 8.0). Cardiac TnI eluted at 40–50% of a linear gradient of 0–0.5 M NaCl in buffer B. If required, as determined by SDS-PAGE, cTnI was dialyzed against buffer B and further purified using a DEAE fast flow column (GE Healthcare). Cardiac TnI was then dialyzed extensively against 5 mM ammonium bicarbonate, lyophilized, and stored at −20°C.

### Determination of Ca^2+^ Binding Affinity by Steady-state Fluorescence Measurements

Fluorescent labeling of cTn^T53C^ and reconstitution of the Tn complexes were prepared as described by Davis *et al.* [21]. All steady-state fluorescence measurements were performed using either a Cary Eclipse fluorescence spectrophotometer (Agilent) or Perkin-Elmer LS55 spectrophotometer at 15°C ±0.1°C. The IAANS fluorescence emission of WT cTnC^T53C^ and L29Q cTnC^T53C^ was measured during Ca^2+^ titration with an excitation wavelength of 330 nm and an emission wavelength of 450 nm. For these experiments the free Ca^2+^ concentration was calculated using EGCA02, a program developed by Robertson and Potter [22].

Microliter amounts of CaCl_2_ were added to 2 mL of each labeled cTn complex (0.15 µM) and reconstituted thin filaments (0.15 µM) in a titration buffer (in mM: 200 MOPS, pH 7.0, 150 KCl, 3 MgCl_2_, 1 DTT, and 2 EGTA) with constant stirring. Each titration measurement was at least duplicated and four to five datasets were collected for each cTn complex for each thin filament preparation. The data were fit with a Hill-equation [23] in Origin 8.5 (Microcal Software, Northampton, MA). The Ca^2+^ affinity for each cTn complex was represented by the dissociation constant K_d_ ± SEM at the half-maximal fluorescence change [23]. Two means were considered to be significantly different when the *p*<0.05.

### Determination of Ca^2+^ Dissociation Rates by Stopped-flow Kinetic Fluorescence Measurements

Reconstituted thin filaments were prepared using protocols described previously [21], [24]. Ca^2+^ dissociation rate constants (k_off_) were measured at 15°C using a stopped-flow apparatus with a dead time of 1.4 ms (model SX. 18 MV, Applied Photophysics, Leatherhead, UK). The rates of conformational changes induced by EGTA removal of Ca^2+^ from labeled cTn complexes or thin filaments were measured. The samples were excited at 330 nm and the emission was monitored through a 420–470 nm band-pass interference filter for the cTn complexes and a 510 broad interference filter for the thin filaments. Each k_off_ value represents an average of at least three separate experiments with each averaging at least five traces fit with a single exponential equation. The buffer for the stopped-flow measurements was in mM: 10 MOPS, 150 KCl, 3 MgCl_2_, 1 DTT, pH 7.0 at 15°C.

### Skinned Cardiac Myocyte Preparations (SCM)

Ventricular myocytes were obtained by enzymatic digestion of hearts from C57BL/6 female mice (3 to 4 mos.) [12]. The experimental protocols involving mice used in this study were in accordance with the Canadian Council on Animal Care (CCAC) regulations. The Animal Care Committee of Simon Fraser University approved the animal protocols used in this study. The cardiac myocytes were subsequently skinned by suspending the cell pellet in relaxing solution (in mM: MgCl_2_ 5.70, Na_2_ATP 6.31, EGTA 2, potassium propionate 156, BES 10, DTT 1, leupeptin 0.1, PMSF 0.1, pH 7.0) containing 0.3% Triton-X for 4 minutes. The myocyte pellet was washed twice using cold relaxing solution. Finally, the SCM were re-suspended in relaxing solution and kept on ice during the day of the experiment.

### Troponin Extraction and Reconstitution in SCM

Cardiac troponin complex substitution was performed using a modification of Chandra *et al.* [12], [25]. The endogenous troponin subunits were extracted by incubating the SCM in an extracting buffer containing a mixture of cTnT and cTnI. In brief, cTnT (1.5 mg/ml, W/V) and cTnI (1.0 mg/ml, W/V) were dissolved in a buffer containing 50 mM Tris-HCl (pH 8.0), 6 M urea, 1.0 M KCl and 10 mM DTT. The salt and urea were then removed by successive dialysis against the same buffer with steadily decreasing KCl and urea concentrations. The mixture of recombinant cTnT and cTnI was finally dialyzed against the extraction buffer containing (in mM:) 10 BES (pH 6.8), 150 KCl, 5 EGTA, 5.7 MgCl_2_, and 1.0 DTT at 4°C. The SCM were bathed in the extraction buffer containing either cTnT-WT cTnI or cTnT-SD cTnI for approx. 20 min at 15°C. The SCM were then washed twice with the relaxing solution. The maximal Ca^2+^-activated force was measured before and after cTnT-cTnI treatment to determine the extent of endogenous cTn removed. A maximal force decrease ≥90% was used as a criterion for cTn extraction, indicating high endogenous cTn [25] extraction. The cTnT-cTnI treated SCM were subsequently reconstituted by incubating in relaxing solution containing related cTnT-cTnI complex plus an excess of either WT or L29Q cTnC (∼4 mg/ml, W/V) for approximately 30 min at 15°C. The maximal Ca^2+^-activated force was measured to determine the extent of cTn reconstitution and SCM with >80% force recovery was used as an inclusionary criterion and was not significantly different between the recombinant cTn complexes.

### Determination of the Ca^2+^ Sensitivity of Force Generation and Length-dependence of Myofilament Ca^2+^ Sensitivity

The apparatus used to measure SL and force from SCM and for rapid solution changes has been described previously [12], [16]. Briefly, a single SCM was attached to a force transducer (Model 406A, Aurora Scientific Inc.) and a length controller (308B, Aurora Scientific Inc.) both of which were controlled by micromanipulators. The myocyte was gravity-superfused using a special three-barrel pipette attached to a fast-step switcher (Warner Instruments, Hamden, CT, USA) for quick solution change. The experimental temperature-controlled chamber was mounted on a stage of a Nikon (Mississauga, ON, Canada) TE2000-U inverted microscope. The temperature of the experimental chamber was controlled at 15°C. SCM were imaged using a Pulnix (Sunnyvale, CA, USA) CCD camera and SL was measured by fast Fourier transform analysis of the digitized striated images of the attached cell [12].

The steady-state force-pCa relationship was determined by measuring the Ca^2+^-activated force (F) with preset SL as the reconstituted myocyte was exposed to a series of solutions with varied Ca^2+^ concentrations (pCa 4.5–8.0) [12], [16]. The length-dependent Ca^2+^ sensitivity was determined in separate experiments by carrying out two force-pCa measurements for each SCM with the SL of the myocytes set to 1.9 µm and subsequently to 2.3 µm.

Each force-pCa curve was fit to the Hill equation using Origin 8.5 as described before [12] to determine the Hill coefficient and *K*_F1/2_ which represents the Ca^2+^ sensitivity and corresponds to the [Ca^2+^] required for generating half-maximal force. The data were analyzed statistically using Student’s *t*-test and one-way repeated measures analysis of variance (One way ANOVA). The values of *K*_F½_ were reported as means ± SEM. Two means were considered to be significantly different when the *p*<0.05.

### Docking and Molecular Dynamics

Docking of the cTnI_1–32_ peptide (PDB: 2JPW) on the WT cNTnC+switch peptide cTnI _147–163_ (PDB: 1MXL) was performed on the ClusPro 2.0 server [26]. Incorporation of the L29Q cTnC mutation, and pseudo phosphorylation of cTnI (Ser23Asp, Ser24Asp) were made manually in COOT [27]. The GROMACS 4.5.4 software suite [28] was used to perform the simulations in an NPT environment using the GROMOS96 53a6 force field.

Each protein system was embedded in a cubic box with 9 Å between the edge of the protein and the edge of the box and solvated using the SPC-E water model. The net charge of the system was made zero by replacing randomly selected solvent molecules with Na or Cl ions. The solvated system was energy minimized using the steepest descents algorithm to an *F*_max_ <1000.0 kJ mol^−1^ nm^−1^ and equilibrated for 1 ns with a time step of 0.002 ps, with position restraints placed on all atoms of the protein and peptide. Interactions were calculated using the Particle Mesh Ewald method [29]. Temperature and pressure coupling were handled by A velocity rescaling thermostat [30] and a Parrinello-Rahman barostat [31] at 300 K using a τ_T_ value of 0.1 and a τ_P_ value of 1.0. The simulation box was periodic in all dimensions. After equilibration, the position restraints on the protein atoms were replaced with P-LINCS bond length constraints and bond angle restraints [32]. Each simulation was run for a total of 100 ns on the Westgrid system “lattice”, followed by steepest descents energy minimization to an *F*_max_ of 250.0 kJ mol^− 1^ nm^− 1^. Analyses of the simulations were carried out using Visual Molecular Dynamics [33] and the GROMACS suite of programs [28]. Figures were prepared using PyMOL [34]. Interhelical angles were calculated using Interhlx [35].

## Results

### Effects of the L29Q Mutation on the Ca^2+^ Binding Affinity of the cTn Complexes and the Reconstituted Thin Filament Preparation (TFP) Determined by Steady-state Fluorescence

The formation and the stability of both the cTn complex and the reconstituted TFP were individually verified using stopped-flow kinetics. The complexes were found to be stable in the absence of free cTnC, and free cTn complexes were not detected in the reconstituted TFP. The abbreviations for the various constructs used in the different preparations are listed in Table 1.

**Figure 1:**
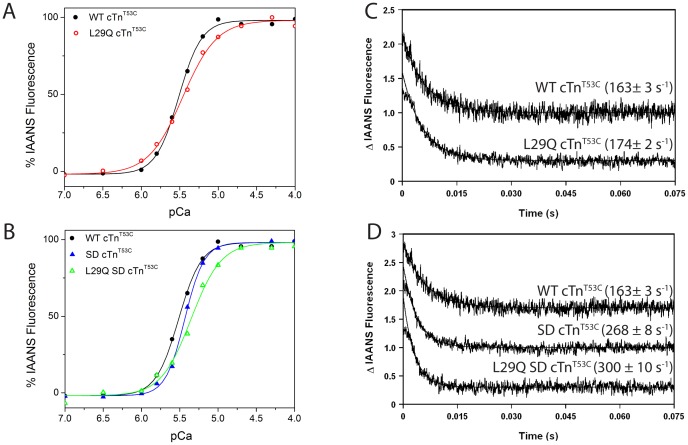
Effects of the L29Q mutation on the Ca^2+^ binding affinity and rate of the Ca^2+^ dissociation from the thin filament (TF). In panels A and B, the ordinate shows the change in fluorescence (% IAANS Fluorescence) in various TF preparations as a function of free Ca^2+^. The data were normalized by setting the fluorescence at pCa 7 equal to 0 and the fluorescence at pCa 3.0 equal to 100% for each preparation. (A) The Ca^2+^-dependent increase in IAANS fluorescence of the thin filament containing the control WT cTnT53C (•) and L29Q cTnT53C (○). (B) The Ca^2+^-dependent increase in IAANS fluorescence of the thin filament containing the control WT cTnT53C (•), SD cTnT53C (▴), and L29Q SD cTnT53C (Δ). In panels C and D, the ordinate shows the change in fluorescence (Δ IAANS fluorescence) in arbitrary units in response to Ca^2+^ removal as a function of time. (C) The time course of the decreases in IAANS fluorescence as Ca^2+^ was removed by EGTA from TF containing the WT cTnT53C (•) and L29Q cTnT53C (○). (D) The time course of the decreases in IAANS fluorescence as Ca^2+^ was removed by EGTA from TF containing the WT cTnT53C (•), SD cTnT53C (▴), and L29Q cTnT53C (Δ).

A measurable Ca^2+^-dependent IAANS fluorescence decrease was observed in all four cTn complexes and no significant differences in the steady-state Ca^2+^ binding or dissociation parameters were observed (Table 2). Changes in Ca^2+^ binding affinity of different cTn complexes became evident upon titrating the reconstituted TFP with Ca^2+^. A Ca^2+^-dependent fluorescence increase was observed for each of the four TFP configurations. The K_d_ of TFP containing the SD cTnI (SD cTn^T53C^) was 1.2 fold higher than TFP containing the WT cTnI (WT cTn^T53C^) (*p*<0.05), indicating that the pseudo-phosphorylated cTnI significantly decreased the Ca^2+^ affinity of cTnC in the TFP. Unexpectedly, the K_d_ of TFP containing L29Q cTnC (L29Q cTn^T53C^) was ∼1.2 fold higher than that of the WT (*p*<0.05), suggesting that TFP containing L29Q cTnC had a lower affinity for Ca^2+^ (Fig. 1 *A*). This is in contrast to the observed increase in Ca^2+^ binding affinity in isolated L29Q cTnC [12]. When plotting fluorescence of TFP that include the pseudo-phosphorylated cTnI (L29Q SD cTn^T53C^) as a function of Ca^2+^ concentration, TFP that include L29Q cTnC produce a curve with a statistically significant rightward shift of 0.08 pCa units relative to that of SD cTn^T53C^ with WT cTnC (*p*<0.05) (Fig. 1 *B*), indicating that L29Q further reduced the Ca^2+^ affinity.

**Table 2:** The Ca^2+^ binding properties of the cTn complex and the reconstituted thin filament preparation (TFP) using the cTnC^T53C^ IAANS* reporter.

Troponin Complex	K_d_ (µM)	Hillcoefficient (n)	k_off_ (s^−1^)
	***cTn complex***		
WT cTn^T53C^	1.01±0.04	0.99±0.06	33.1±1.4
SD cTn^T53C^	0.98±0.07	0.91±0.04	32.7±0.3
L29Q cTn^T53C^	1.07±0.06	0.93±0.05	32.2±0.6
L29Q SD cTn^T53C^	0.99±0.08	0.93±0.02	33.2±0.2
	***Thin filament preparation***		
WT cTn^T53C^	3.05±0.06	2.94±0.23	163±3
SD cTn^T53C^	3.71±0.27^a^	3.66±0.29	268±8^a^
L29Q cTn^T53C^	3.51±0.04^a^	1.93±0.29^*a*^ ^***b***^	174±2^b^
L29Q SD cTn^T53C^	4.53±0.38^*a*^ ^***b***^	2.25±0.22^b^	300±10^*a*^ ^***b***^

* cTnC^T53C^: three mutations (C35S, C84S, and T53C) are introduced into native cTnC for IAANS labelling at the T53C position [21].

^*a*^Significantly different from their respective WT control values (*p*<0.05, unpaired t-test versus WT cTn).

^***b***^Significantly different from their respective SD values (*p*<0.05, unpaired t-test versus SD cTn).

Table 2 also shows the Hill coefficients of the cTn complexes and the reconstituted TFP. The Hill coefficients (*n*_H_) of all the cTn complexes were within the range of 0.91–0.99 and no significant differences were observed among the different cTn complexes. The values of the Hill coefficients of the TFP were, as anticipated, much higher overall. The Hill coefficient of the TFP containing WT cTn was significantly higher when the pseudo-phosphorylated SD cTnI was included in the complex. The presence of the L29Q cTnC mutation significantly (*p*<0.05) decreased the Hill coefficient by 34% and 23% for the WT cTnI and SD cTnI containing complexes, respectively. These data reveal that the L29Q mutation has an effect on Ca^2+^ regulation in TFP containing either the WT or the phosphomimetic cTnI.

### Effects of the L29Q Mutation on the Ca^2+^ Dissociation Rates from the Reconstituted Thin Filaments Determined by Stopped-flow Fluorescence Measurements

Examination of the effect of phosphomimetic TnI mutations in the context of TFP yielded a Ca^2+^ k_off_ value 1.6 fold higher than that observed for WT (*p*<0.05). In TFP containing WT cTnI, the inclusion of L29Q cTnC did not appear to have any effect; however in SD TnI containing TFP, the presence of the L29Q mutation further increased the Ca^2+^ dissociation rate concomitant with the effect of the pseudo-phosphorylated cTnI.

### Troponin Exchange in Skinned Cardiomyocytes

Due to the difficulty in running analytical PAGE on single skinned cardiac myocytes, the efficacy of native cTn complex exchange with the recombinant cTn complex was determined by measuring the maximal Ca^2+^ activated force changes before and after extraction with the cTnI·cTnT complex. Chandra *et al.* observed a Ca^2+^ activated force lost at 85–90% in detergent skinned left ventricular papillary muscle fiber bundles after cTnI·cTnT treatment. By running analytical PAGE on these skinned fiber bundles, they estimated that at least 80% of the endogenous cTnI was extracted [25]. Our results indicated a similar activated force lost and higher force recovery rate after cTnI·cTnT treatment and reconstitution with various troponin complexes.

### Effects of the L29Q Mutation on the Ca^2+^ Sensitivity of Force Generation in SCM

**Figure 2:**
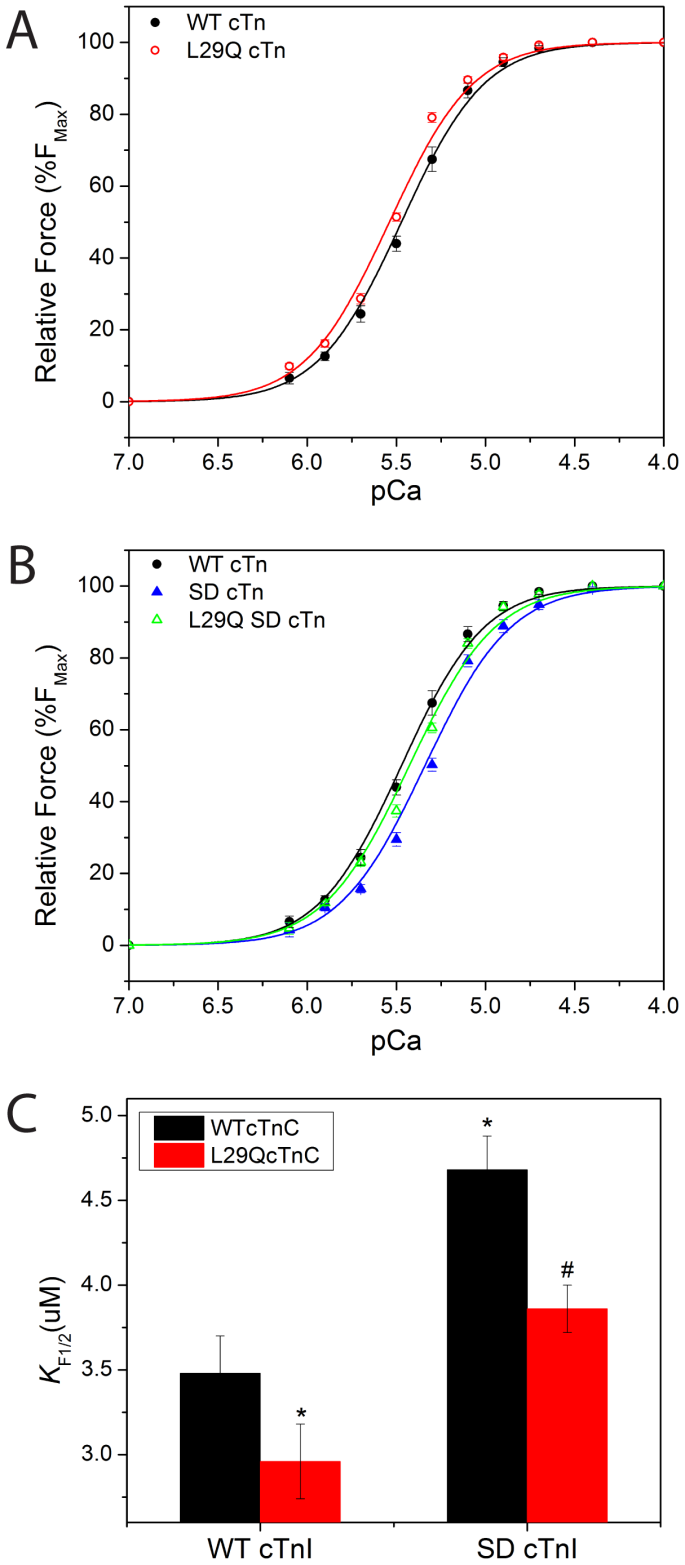
Effects of the L29Q cTnC mutation on the Ca^2+^ sensitivity of force generation in myocytes reconstituted with recombinant cTn containing either WT cTnI or SD cTnI. (A) The comparison of the force-pCa relationships of skinned cardiac myocytes containing either WT cTn or L29Q cTn at SL 2.1 µm. (B) The comparison of the force-pCa relationships of skinned cardiac myocytes containing WT cTn, SD cTn, and L29Q SD cTn. The data are expressed as normalized force, which was calculated as a function of the force generated at pCa 4.5. Data points are means ± SEM of six myocytes at 15°C, pH 7.0. (C) The comparison of KF1/2 values, which is the derived [Ca^2+^] at half-maximal force. The asterisk (*) indicates p<0.05 versus the WT cTn, and the pound sign (#) indicates p<0.05 versus the SD cTn.

The effects of PKA-dependent phosphorylation of the N-terminus of cardiac troponin I (cTnI) on myofilament Ca^2+^ sensitivity have been studied extensively [10], [17], [36], [37]. It has been reported that animal sacrifice can lead to persistence of PKA-dependent phosphorylation of cTnI after the skinning procedure, and this could produce confounding results [13], [38]. In the present experiments, endogenous cTn was replaced with either wild type (WT cTn) or mutant cTn that contained the L29Q cTnC mutation (L29Q cTn). The force-pCa relationships were first determined at a SL of 2.1 µm. Fig. 2*A* shows the relationship between steady-state force development and the free [Ca^2+^] (pCa) in SCM reconstituted with either WT cTn or L29Q cTn. The force-pCa curves of SCM reconstituted with L29Q cTn were left shifted, indicating an increase in myofilament Ca^2+^ sensitivity. The Ca^2+^ sensitivity for L29Q cTn increased 1.2-fold (*p*<0.05) in SCM (Table 3). This finding corroborated our results obtained from SCM in which the endogenous cTnC was replaced with recombinant L29Q cTnC increasing Ca^2+^ sensitivity 1.4-fold compared to WT cTnC [12].

**Table 3:** Myofilament Ca^2+^ sensitivity determined in single skinned cardiac myocytes (SCM) reconstituted with various recombinant Tn complexes at sarcomere length of 2.1 µm.

SCMReconstitution	K_F1/2_(µM)	ΔK_F1/2_	Hillcoefficient
Tn complex (N)	Mean ± SEM	(µM)^c^	(n)
WT cTn (7)	3.40±0.11		2.08±0.02
L29Q cTn (7)	2.95±0.07^a^	−0.45	2.12±0.02
SD cTn (6)	4.68±0.20^a^	+1.28	2.16±0.04
L29Q SD cTn (6)	3.86±0.14^b^	+0.38	2.09±0.03

a significantly different from their respective WT control value (*p*<0.05, unpaired t-test versus WT cTn).

b Significantly different from their respective SD values (*p*<0.05, unpaired t-test versus SD cTn).

c ΔK_F1/2_ = K_F1/2_ of various cTn−K_F1/2_ of WT cTn.

### Effects of the cTnC^T53C^ IAANS Reporter on Experiments Performed in SCM

Because of the difference in the results seen in TFP in which the cTnC^T53C^ IAANS reporter was used and SCM experiments in which no reporter was used, control experiments were performed in SCM. In these experiments, cTnC^T53C^ IAANS reporter was used in additional reconstitution experiments of SCM with the WT cTnC and L29Q cTnC constructs (Table 4). Three observations were made: 1) when SCM were reconstituted with recombinant troponin complexes without the reporter, a minimum of 80% force recovery was used as an inclusionary criterion. With the troponin complexes containing the cTnC^T53C^ IAANS reporter, the criterion had to be reduced as incorporation into SCM was consistently lower. In this study, the maximum force recovery with the reporter averaged ∼75%. 2) Reconstitution of SCM with troponin complexes incorporating the cTnC^T53C^ IAANS reporter had a slight but significant effect of Ca^2+^ desensitization. As can be seen in Table 4, both WTcTn^T53C^ IAANS and L29QcTn^T53C^ IAANS had significantly higher K_F1/2_ values than their respective non-reporter controls. 3) In the comparison of the L29Q cTnC with and without the IAANS reporter in SMC, the L29Q mutation consistently had a Ca^2+^ sensitizing effect and the L29QcTn^T53C^ IAANS exhibited significantly (p<0.05) lower mean K_F1/2_ values than WT cTn ^T53C^IAANS (Table 4).

**Table 4:** Myofilament Ca^2+^ sensitivity determined in single skinned mouse cardiomyocytes (SCM) reconstituted with recombinant Tn complexes ± cTn^T53C^ IAANS reporter at sarcomere length 2.1 µm.

SCM reconstitution	K_F1/2_ (µM)		Difference between groups		
Tn complex (N)	Mean ± SEM		K_F1/2_ (µM)	SEM	P Value
WTcTn (7)WT cTnCT53C (5)	3.40±0.113.86±0.09	}	+0.46	0.14	0.013^a^
L29QcTn (7)L29Q cTnT53C (6)	2.95±0.073.40±0.10	}	+0.45	0.13	0.013^b^
WT cTnC^T53C^(5)L29Q cTnT53C (6)	3.86±0.093.40±0.10	}	−0.46	0.14	0.017^c^

ΔK_F1/2_ = K_F1/2_ of various cTn in bottom row of each pair- K_F1/2_ of top row of each pair.

### Effects of the L29Q cTnC Mutation on the Ca^2+^ Sensitivity of Force Generation in SCM Reconstituted with Recombinant cTn Containing SD cTnI

After replacing the endogenous cTn with recombinant cTn that contained WT cTnC and SD cTnI (SD cTn) in SCM, the force-pCa curves were shifted to the right of those of SCM reconstituted with recombinant WT cTn which included WT cTnC and WT cTnI (Fig. 2 *B*). The Ca^2+^ sensitivity of force generation of SCM reconstituted with SD cTn was 1.38-fold lower than that of SCM with WT cTn. These results are consistent with previous findings which showed that phosphorylation of Ser23/24 reduced the Ca^2+^ affinity of cTnC thereby increasing the rate of muscle relaxation [39], [40]. Interestingly, the Ca^2+^ sensitivity of force generation for SCM containing either WT cTnI or SD cTnI was increased when L29Q cTnC is present (shown by the decreased K_F1/2_ value, Fig. 2*C*, Table 3). In summary, at a SL of 2.1 µm, SCM containing SD cTnI showed a significantly decreased Ca^2+^ sensitivity, and this reduction of Ca^2+^ sensitivity was diminished by approximately 68% by the presence of the L29Q cTnC mutation.

### Effects of L29Q cTnC on the Sarcomere Length-dependence of Myofilament Ca^2+^ Sensitivity in SCM Reconstituted with cTn Containing SD cTnI

**Figure 3:**
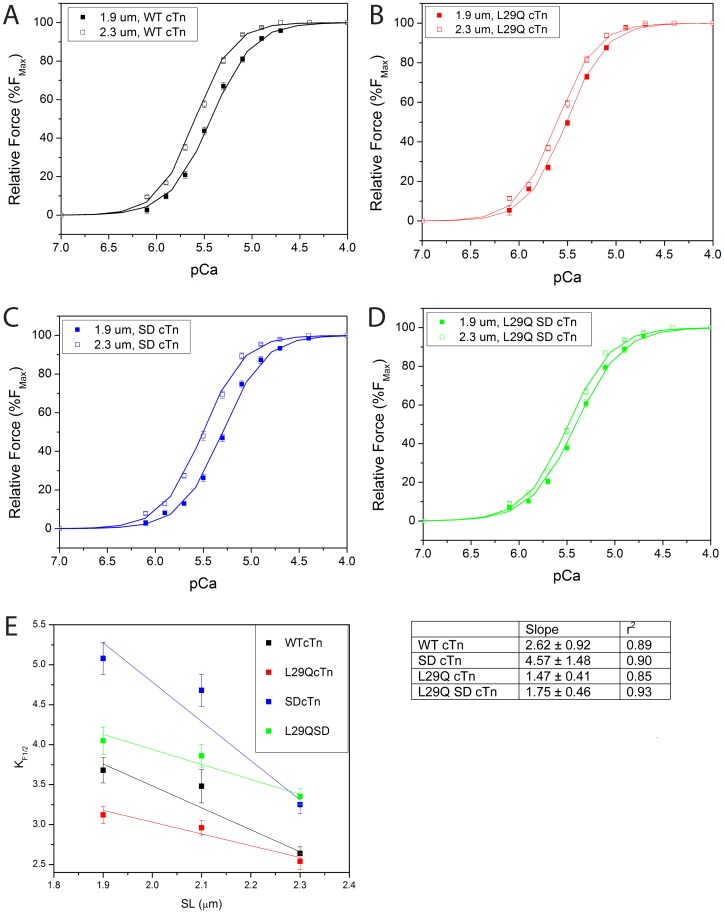
Effects of the mutant L29Q cTnC and SD cTnI on length-dependent Ca^2+^ sensitivity in skinned cardiac myocytes. The changes of force-pCa relationship as sarcomere length (SL) was increased from 1.9 to 2.3 µm in the skinned myocytes reconstituted with (A) WT cTn, (B) L29Q cTn, and (C) SD cTn, and (D) L29Q SD cTn. Data points are means ± SEM of 6 myocytes at 15°C, pH 7.0. (E) Ca^2+^ sensitivity of force generation as a function of SL. The KF1/2 values (in µM) for SL 2.1 µm are from the experiments shown in Fig. 2. The data were fit to a linear regression line. The table shows the values of r2 and resultant slopes (µM/µm) for myocytes reconstituted with each construct.

Fig. 3 shows the relationship between steady-state force development and pCa in SCM reconstituted with WT cTn (Fig. 3*A*), SD cTn (Fig. 3*B*), L29Q cTn (Fig. 3*C*) and L29Q SD cTn (Fig. 3*D*) with SL clamped at either 1.9 µm or 2.3 µm. For all groups of SCM, the force-pCa curves were shifted to the left at the greater SL, indicating an increase in myofilament Ca^2+^ sensitivity with sarcomeric stretch. These results corroborate the well-documented length-dependent myofilament Ca^2+^ sensitivity [41]. As shown in Fig. 3*E*, the K_F1/2_ values for the SD cTn at both SL 2.3 µm and 1.9 µm were significantly higher than that for the WT cTn at both SL (*p*<0.05), indicating that SD cTnI rendered the SCM less sensitive to Ca^2+^. This phenomenon is more pronounced at shorter SL (1.9 vs. 2.3 µm), demonstrating a significant increase in the length-dependent myofilament Ca^2+^ sensitivity. In addition, the SCM reconstituted with L29Q SD cTn had a higher K_F1/2_ at SL 1.9 µm that was significantly different from both WT and SD (*p*<0.05); however, at SL 2.3 µm, the K_F1/2_ of L29Q SD cTn was significantly different from WT (*p*<0.05) but was not different compared to SD cTn. This resulted in a much shallower slope of the K_F1/2_ value and SL relationship in SCM containing L29Q SD cTn than those containing SD cTn and WT cTn (Fig. 3*E*, Table 5), indicating that LDA was significantly reduced by the L29Q cTnC mutation.

**Table 5:** Myofilament Ca^2+^ sensitivity (K_F1/2_) and Hill coefficients (n) in SCM as a function of sarcomere length.

SCM Reconstitution	K_F1/2_ (µM)		K_F1/2_	Hill (n)	
Tn Complex (N)	1.9 µm	2.3 µm	fold ↑	1.9 µm	2.3 µm
WT cTn (5)	3.68±0.16	2.64±0.08	1.39	2.03±0.08	2.09±0.08
SD cTn (6)	5.08±0.21^a^	3.25±0.12^a^	1.56	2.07±0.05	2.01±0.06
L29Q cTn (6)	3.12±0.11^*ab*^	2.09±0.04	1.49	2.54±0.10	2.08±0.02
L29Q SD cTn (6)	4.05±0.17^*ab*^	3.35±0.10^a^	1.21	2.10±0.07	2.08±0.03

Data are given as mean ± SEM.

^*a*^Significantly different from their respective WT control value (*p*<0.05, unpaired t-test versus WT cTn).

^*b*^Significantly different from their respective SD values (*p*<0.05, unpaired t-test versus SD cTn).

K_F1/2_ fold ↑ = K_F1/2_ measured at 1.9 µm/K_F1/2_ measured at 2.3 µm.

### Docking and Molecular Dynamics Simulations (MDS) of the Cardiac Specific N-terminus of cTnI on the Binary Complex of the cNTnC and Switch Peptide of cTnI

**Figure 4:**
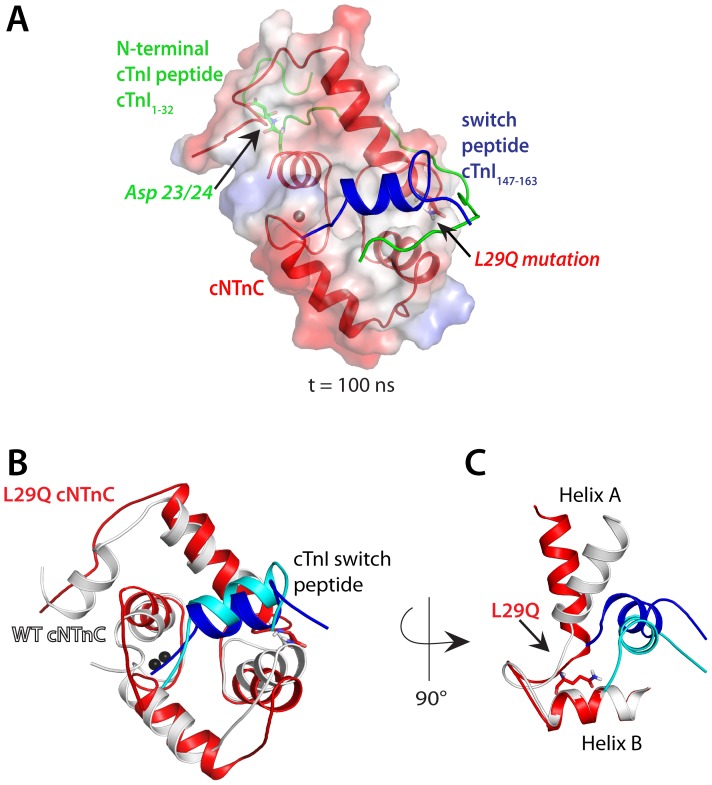
Effects of the mutant L29Q cTnC and SD cTnI determined by Molecular Dynamics simulations. (A) MDS-based model for the interaction of the cardiac specific N-terminal extension of cTnI peptide1–32 (green; PDB: 2JPW) with the pseudo-phosphorylated Asp23/24 indicated. The N-terminus of L29Q cTnC is shown in red and cTnI switch peptide147–163 is shown in blue (PDB: 1MXL) with the L29Q mutation indicated. Electrostatic surface of the N-terminus of L29Q cTnC was generated in pyMOL using APBS with 50% transparency (red, negatively charged; blue, positively charged; white, neutral). This is at t = 100 ns of simulation. (B) WT cNTnC (white) is superimposed with L29Q cNTnC (red) with its cTnI switch peptide bound to its central hydrophobic cavity at t = 100 ns simulation. (cyan: cTnI switch peptide bound within WT cNTnC; dark blue: cTnI switch peptide bound within L29Q cNTnC). The pseudo-phosphorylated cTnI is omitted from this snapshot for clarity. The Ca^2+^ ion bound in site II is shown as a black sphere. (C) shows the largest movement caused by the L29Q mutation in the presence of the pseudo-phosphorylated cTnI, which is the re-orientation of helix A and helix B of cNTnC accompanied by a decrease of interhelical angle of 13°.

To investigate the structural and dynamic changes in cNTnC as a result of the L29Q mutation, we performed MDS of WT and L29Q cNTnC with the switch peptide of cTnI_147–163_ (PDB: 1MXL), docked with the cardiac specific N-terminal extension of cTnI_1–32_ (PDB: 2JPW) with and without pseudo-phosphorylation (Fig. 4*A*). The docking position of the N-terminal extension of cTnI_1–32_ on cNTnC was manually adjusted based on the intermolecular interactions suggested by Howarth et al. [42], taken together with cNTnC chemical shift perturbation data, which suggested that the binding location of cTnI_1–32_ was near the β-sheet region of cNTnC, separate from the hydrophobic cavity where the cTnI_147–163_ switch peptide binds [8], [11].

After 100 ns of simulation, the major structural changes within the triplex were the position of the cTnI switch peptide and the N-terminal extension of cTnI_1–32_ with respect to the cNTnC molecule. The cTnI N-terminus assumes a different location in each of the four simulations, indicating variability in the binding of the cTnI N-terminus. The simulated structure with the lowest potential energy is shown in Fig. 4 *A*. Interhelical angle analysis between helix A and helix B was performed to investigate the opening/closing of the cNTnC conformation when cNTnC is bound to Ca^2+^ and the cTnI switch peptide [43]. Throughout the 100 ns simulation, un-phosphorylated cTnI_1–32_ in the WT complex demonstrated the greatest flexibility, particularly toward the C-terminal end of cTnI_1–32_. Serines 23 and 24 do not interact with any residues of cNTnC and are pointed outward toward the bulk solvent; this agrees with the hypothesis that these serine residues must be solvent accessible in order to be phosphorylated by protein kinases during β-adrenergic stimulation [42]. During the simulation of the WT complex, the interhelical angle between helices A and B was increased from 100° to 106° when cTnI_1–32_ was pseudo-phosphorylated, suggesting pseudo-phosphorylation of cTnI promotes a less open conformation in cNTnC. In complexes containing the L29Q mutation, the angle between helix A and helix B decreased from 95° to 93° when cTnI is pseudo-phosphorylated, suggesting that the L29Q cNTnC adopts a similar conformation regardless of pseudo-phosphorylation. The interhelical angles for L29Q simulated structures were 13° smaller than those of WT when cTnI is pseudo-phosphorylated, showing that L29Q mutation caused a significantly more “open” conformation of cNTnC in the presence of pseudo-phosphorylated cTnI (Fig. 4 *B* and 4 *C*).

## Discussion

In the comparison of the effect of the L29Q cTnC mutation in isolated Tn complexes, neither the Ca^2+^ binding affinity nor the Ca^2+^ dissociation rates were different between the WT cTn^T53C^ and L29Q cTn^T53C^ complexes. Similarly, no significant difference in Ca^2+^ binding affinities or kinetics of isolated Tn complexes containing pseudophosphorylated cTnI and either TnC^T53C^ or L29Q cTnC^T53C^ were observed. While these results were not entirely expected, they suggest that a more complex system that includes both actin and tropomyosin is required to study the interactions between troponin subunits [24]. A similar trend has been observed for other cardiomyopathy-related cTnI and cTnT mutations that had minimal effect on the cTn complex level while demonstrating larger sensitizing or desensitizing effects on the reconstituted thin filament [44]. In reconstituted thin filaments, the L29Q cTnC mutation did not change the rate of Ca^2+^ dissociation from cTn though it caused a small but significant decrease in Ca^2+^ binding affinity. However, in TFP reconstituted with SD cTnI, the presence of the L29Q mutation further enhanced the increase of the Ca^2+^ dissociation rate caused by pseudo-phosphorylation of cTnI. These results convincingly demonstrated that at the thin filament level, the L29Q cTnC mutation alters the ability of Ca^2+^ binding to cTnC and affects the functional effects of the phosphomimetic cardiac troponin I. It has been well-documented that phosphorylated cTnI decreases the Ca^2+^ binding affinity of cTnC by increasing the k_off_ to facilitate muscle relaxation [39], [40], [45], [46]. The L29Q cTnC mutation seems to exert its effect, at least in part, by perturbing the interaction between the cTnI N-terminus and the switch region [11]. It was shown that the G159D cTnC mutation blunts the impact of cTnI Ser23/24 pseudo-phosphorylation on myofilament function in skinned rat trabeculae [47].

To understand better the effect of the L29Q cTnC mutation in a system closer to physiological conditions, the native cTn complexes were exchanged with various recombinant cTn complexes containing both the L29Q and WT cTnC in SCM. The decrease in Ca^2+^ binding affinity resulting from the L29Q cTnC mutation observed in the thin filament preparation was not commensurate with a reduction in Ca^2+^ sensitivity of force generation in SCM. Instead, the L29Q cTnC mutation resulted in a significant increase in Ca^2+^ sensitivity of myofilament activation (similar to what we observed previously with cTnC reconstitution [12]) and correspondingly reduced the Ca^2+^ desensitization effect of SD cTnI. One of the possible reasons for these divergent results may be related to the effects of the cTnC mutations (C35S/T53C/C84S) designed for the incorporation of the cTnC^T53C^ IAANS or the IAANS itself we used as a Ca^2+^ reporter in the *in vitro* experiments on TFP in comparison to the experiments without the IAANS reporter in the reconstituted SCM. Previous studies have shown that these cTnC mutations and subsequent labeling with IAANS behave biochemically and physiologically similar to wild-type and endogenous cTnC [21]. Our present data (Table 4) using SCM reconstituted with cTnT^53C/IAANS^ demonstrate that while the engineered reporter is not completely innocuous on SCM function, the sensitizing effect of the L29Q cTnC has approximately equal magnitude of −0.46 and −0.45 µM in the presence and absence of the IAANS reporter’ respectively. Thus the data support the notion that the differences in observations on the effect of L29Q cTnC between the thin filament preparation and the skinned cardiomyocyte are distinct from the uniform sensitizing effect of the cTnC^T53C/IAANS^ reporter that is observed in the WT and L29Q experiments.

Two other major differences between TFP and SCM that could contribute to the differences in results we obtained are the impact of: sarcomere length and the presence of myosin and other proteins of the thick filament. While these are not completely mutually exclusive concepts, SL will be discussed first.

Our previous study using the cTnC exchange technique in the skinned cardiomyocytes [12] was the first to demonstrate that increased Ca^2+^ sensitivity of force induced by the L29Q cTnC mutation affected length dependent activation (LDA). The present study in which SCM were reconstituted with troponin complexes corroborates that finding. Thus it is important to note that the effect of the L29Q cTnC mutation on Ca^2+^ sensitivity of force generation was more pronounced at shorter SL (1.9 µm) than at longer SL (2.3 µm) (Fig. 3, *A–D*), which resulted in a significant decrease in the slope of the relationship between Ca^2+^ sensitivity of force and the SL (Fig. 3 *E*). Interestingly, a similar reduction in SL-dependence of contractile activation caused by enhanced Ca^2+^ binding of cardiac troponin was also recently reported by Korte *et al.* [48]. They found that another cTnC mutation, L48Q, enhanced Ca^2+^ affinity and reduced the SL-dependence of myofilament Ca^2+^ sensitivity. This finding has several important implications: 1) the L29Q cTnC mutation should impact LDA that is an important component of the Starling relationship of the heart; 2) the high myofilament Ca^2+^ sensitivity of the L29Q cTnC mutation at the shortest SL (1.9 µm) which corresponds to near peak systolic conditions could alter Ca^2+^ dynamics within the cardiomyocyte with the potential of being arrhythmogenic [49]–[51]; and 3) the strong impact of the L29Q cTnC on the LDA could also be an explanation for the discrepant results found in this study between the data from TFP and that from SCM. Furthermore, it may also provide an explanation for the difference in results between our previous ([12] and present study) which showed an increase in Ca^2+^ sensitivity with the L29Q cTnC mutation and others which did not [8], [11], [14]. The *in vitro* studies provide no means of controlling or assessing the length or the conformation of the thin filament which could clearly mask such findings. The multicellular skinned fiber preparations likely pose a problem due to heterogeneity of SL within the preparation and lower values of reconstitution with the recombinant troponins compared to single cardiomyocytes used in the present study. The latter implies higher retention of endogenous cTnC or troponin complex. In the present study, the L29Q cTnC mutation induced a significant increase in Ca^2+^ sensitivity in SCM reconstituted with the recombinant mouse cTn at sarcomere lengths of 1.9 and 2.1 µm but not 2.3 µm. However, by replacing endogenous cTnC with recombinant human L29Q cTnC or WT cTnC in skinned fiber bundles from left ventricular papillary muscle, Dweck *et al.* [10], Neulen *et al.* [13], and Gollapudi *et al.* [14] did not observe the L29Q cTnC mutant to increase myofilament Ca^2+^ sensitivity. Gollapudi *et al*. reported a small but significant decrease in myofilament Ca^2+^ sensitivity by exchanging the endogenous cTn with homologous recombinant cTn [14]. It should be noted, that although Dweck *et al.* [10] found the changes in the Ca^2+^ sensitivities in skinned fibers reconstituted with L29Q cTnC were statistically insignificant with respect to the WT, they did observe tendencies to increase the Ca^2+^ sensitivity of the mutant that led them to conclude that the disparity was attributable to the increased sensitivity of the single SCM system, compared to their skinned multi-cellular cardiac muscle fiber technique. Indeed, single SCM have distinct advantages over skinned cardiac muscle fiber bundles. The shorter diffusion distances in skinned cardiomyocytes will make troponin subunit exchange more effective and avoid the concentration gradient build-up of substrates (ATP) and end products (P_i_ and ADP) during contraction [2]. In our SCM experiments, ∼85% maximal force recovery was observed after extraction of endogenous cTn and reconstitution with exogenous cTn. This value is significantly higher than those reported by Dweck *et al.* (∼70%) [10] and Neulen *et al.* (∼60%) [13]. Higher maximal force recovery implies that more recombinant cTn was successfully reconstituted into the fiber preparation, and there was significantly less residual native cTnC which is important in detecting small changes in myofilament Ca^2+^ sensitivity caused by the cTnC mutants. More importantly, cardiomyocytes are short and thin enough for one to precisely determine SL changes, which is a crucial parameter to be controlled in determining the effects on myofilament Ca^2+^ sensitivity. Thus, the discrepancies between our study and the aforementioned studies may be due to the differences in SL when determining Ca^2+^ sensitivity in skinned cardiac preparations. The study of Gollapudi *et al.* [14] set the SL of skinned cardiac papillary muscle fiber bundle at 2.2 µm while the results from Dweck *et al.* [10] and Neulen *et al.* [13] do not include SL data.

While length dependent activation is an important physiological principle, particularly in heart muscle, considerable controversy as to the mechanism by which LDA increases myofilament Ca^2+^ sensitivity exists [41], [52]–[54]. The prevailing hypothesis for LDA has been attributable to reduced myofilament lattice spacing (MLS) at longer SL [55]–[57] increasing the possibility of cross bridge formation due to proximity. Thus for a given concentration of cytosolic Ca^2+^, one would expect an increased probability of strong cross bridge formation and hence increased myofilament Ca^2+^ sensitivity. Experiments on myofilaments using high molecular weight compounds (e.g. dextran or mannitol) as a means of increasing osmotic compression and causing a reduction in myocyte diameter have demonstrated increased Ca^2+^ sensitivity [58], [59]. Recently however, several studies have challenged the myofilament lattice spacing hypothesis as an explanation for LDA. In particular, experiments using x-ray diffraction to directly measure MLS under a variety of conditions have raised serious doubts about the generality of this hypothesis [41], [54], [55], [60]. Konhilas *et al.* [60] found that muscle fiber diameter was not an absolute indicator of MLS. More recently, studies by Farman *et al.* [54], [55] suggested that LDA arises primarily from length-dependent factors that modulate Ca^2+^ cooperativity including: the influence of length on the interaction between adjacent troponin-tropomyosin complexes on the thin filament [55] or myosin head orientation before activation [54]. Thus with controversy surrounding the mechanisms of LDA, it is somewhat speculative to propose a mechanism by which the L29Q cTnC mutation significantly attenuated LDA in SCM in our experiments. However, we speculate that the effects of the L29Q cTnC mutation on the interactions between the thin filament proteins alter the feedback effects of cross-bridge binding to actin on Tn/Tm complex, resulting in a higher Ca^2+^ sensitivity of force generation. The presence of the other myofilament proteins, such as myosin, that causes the feedback effects of cross-bridge is likely another reason for the different effects of the L29Q cTnC mutation in our reconstituted TFP and SCM.

The above hypothesis is supported, at least in part, by the changes in the Hill coefficients. Studies have shown that the activation of myocardial contraction is highly cooperative, and the alterations in cooperativity are typically, but not always, manifested as a change in the Hill coefficients [2]. Our present data show that there was a significant decrease in the Hill coefficient caused by the L29Q cTnC mutation in the thin filament preparation (Table 2) but not in SCM (Table 3), suggesting that the effect of L29Q cTnC mutation on the cooperativity of thin filament Ca^2+^ activation was altered by the effect of cross-bridges. The presence of other myofilament proteins that cause the feedback effect of bound cross-bridges on L29Q cTnC-cTnI-Tm complex might also play an important role in determining the Ca^2+^ sensitivity of force generation in SCM.

The L29Q cTnC mutation seems to perturb the interaction between the cTnI N-terminus and the cTnI switch region and eventually exerts its effect on the interaction between cTnI and actin. This effect may disturb the feedback effects of cross-bridges on the interaction between cTnC-cTnI-Tm complex and actin. Studies have shown that cross-bridge binding to the thin filaments can increase the apparent affinity of cTnC for Ca^2+^ in cardiac muscle. Although the reduction in SL-dependence of contractile activation caused by the L48Q cTnC mutation in the recent study by Korte *et al.* [48] is likely to be independent of strong cross-bridges, their results also suggest that the alteration in cTnC-cTnI interaction affects the SL dependence of myofilament Ca^2+^ activation in cardiac muscle and corroborates our hypothesis.

Several studies have postulated that the major impact of the L29Q cTnC mutation is on its effect on the interaction of PKA-phosphorylated cardiac troponin I (cTnI) with cTnC and its ability to impact thin filament regulation [8], [11]. Our study corroborates this finding and sheds more light on its possible mechanism. SL dependence of myofilament Ca^2+^ activation has been shown to be affected by PKA-mediated phosphorylation of cTnI. In the present study, SD cTnI caused an expected reduction in Ca^2+^ sensitivity of force generation with a more pronounced and significant decrease in Ca^2+^ sensitivity at SL 1.9 µm than at 2.3 µm. Konhilas *et al.* [61] demonstrated that PKA-mediated phosphorylation of cTnI induces an increase in SL-dependent activation. Interestingly, they found that the Ca^2+^ desensitization was also greater at shorter (1.9 µm) SL, resulting in a steeper relationship between Ca^2+^ sensitivity of force and the SL [61]. The L29Q cTnC mutation exerted its effect on Ca^2+^ sensitivity of force more profoundly at shorter SL, which reduced the increase in the slope of Ca^2+^ sensitivity-SL relationship caused by SD cTnI. These results further support our hypothesis that the alterations in cTnC-cTnI interaction cause more pronounced changes in Ca^2+^ sensitivity of force at shorter SL, thus affecting the length dependence of myofilament Ca^2+^ activation.

In contrast to our results, Rao *et al.* found that S23/24D cTnI reduced pCa_50_ at both SL, primarily at 2.3 µm, resulting in a reduced SL-dependence of Ca^2+^ sensitivity. The source of this discrepancy is not clear; however, it is likely due to the experiment conditions, heterogeneous proteins, and the difference between the skinned cardiac muscle preparations used for the studies as discussed below. Previous studies have shown similar inconsistent results when an increase in SL dependence of Ca^2+^ sensitivity was found in PKA treated skinned cardiac myocyte preparations whereas a decrease was reported in skinned cardiac trabeculae phosphorylated by PKA treatment.

Neulen *et al.* [13] did not find the L29Q mutation to have a significant influence on the PKA-induced Ca^2+^-desensitization, when assayed by incubating the cardiac muscle fiber bundles with phosphatase or PKA to phosphorylate sarcomeric PKA target proteins (e.g. N-terminus of cTnI, cardiac myosin-binding protein C (MyBP-C), and titin). PKA phosphorylation of the N-terminus of cTnI and MyBP-C may suppress cardiac contractility [62]. Also, PKA phosphorylation of titin results in decreases in passive force, and titin based passive tension changes impacts Ca^2+^ sensitivity in skinned rat ventricular trabeculae. Therefore, the phosphatase and PKA treatments of sarcomeric PKA substrates in skinned cardiomyocytes might affect Ca^2+^ sensitivity of force generation masking the effects of the L29Q mutation on Ca^2+^ activation.

Our MDS data demonstrate further the changes of the cNTnC conformation due to the L29Q cTnC mutation (Fig. 4). In the presence of pseudo-phosphorylated cTnI, the smaller opening of helix A and helix B of WT cNTnC suggests that the pseudo-phosphorylated cTnI reduces the affinity of cNTnC for the cTnI switch peptide. This is due to the closed conformation of cNTnC and potentially destabilizes the binding of cTnI to the hydrophobic cavity of cNTnC. This supports the previous notion that phosphorylation of Ser23/24 of cTnI facilitates the Ca^2+^ release from cNTnC thereby aiding the muscle relaxation process (11). Conversely, in the presence of the L29Q cTnC mutation, the pseudo-phosphorylation of cTnI does not cause any significant change in the opening/closing of cNTnC. The L29Q cTnC mutation renders the cNTnC in a more “open” conformation compared to the constructs containing WT cTnC especially when cTnI is pseudo-phosphorylated (Figs. 4 B and C). Though Leu29 does not make key contacts with residues to form the hydrophobic core of cNTnC, such as in the L48Q cTnC mutant (49), the L29Q cTnC mutation does have a small global effect on the conformation of cNTnC. The L29Q cTnC mutations promotes a more open conformation that could have increased the affinity of the cTnI switch peptide for cNTnC, reduced its inhibitory function on actin and impeded the muscle relaxation process.

## Conclusions

The L29Q cTnC mutation decreased the Ca^2+^ binding affinity and enhanced the effects of pseudo-phosphorylation of cTnI in thin filament preparations but caused a small yet significant increase in Ca^2+^ sensitivity of force and reduced the effects of pseudo-phosphorylation of cTnI in single SCM. More importantly, the effect of the L29Q cTnC mutation on Ca^2+^ sensitivity of force generation is extremely SL-dependent with the impact being maximal at the shorter SL (1.9 µm) and is virtually abolished at longer SL (2.3 µm). In other words, the L29Q cTnC mutation significantly blunted the length-tension relationship in this preparation which likely has consequences on the Frank Starling relation *in vivo*. Very recently this was also observed in a study using a wide variety of hypertrophic cardiomyopathy mutations in humans [63] thus corroborating our previous finding [12] and supporting the present conclusions. The mutation also depressed the SL-dependent increase in Ca^2+^ sensitivity in the presence of pseudo-phosphorylation of cTnI. The effects of the L29Q mutation on Ca^2+^ sensitivity of force may be caused by its interference on the interactions between thin filament proteins which alter the feedback effects of cross-bridges on the cTnC-cTn/Tm complexes. The small increase in myofilament Ca^2+^ sensitivity might not alter the contractility of cardiac muscle significantly during the daily activities of the patients with this cTnC mutation. However, during β-adrenergic stimulation where increased cardiac output is required, the mutation might affect pump function by reducing the length dependence of Ca^2+^ activation and the response of cardiac troponin to increased cardiac output. These effects are likely hypertophic in response to defects in contractility when physiological demand is increased. Our results are consistent with the postulate [10] that the Ca^2+^ sensitizing effect of FHC-related cTnC mutants becomes more pronounced in the presence of PKA-mediated phosphorylation of cTnI induced by β-adrenergic stimulation but this effect is clearly sarcomere length dependent.
